# Clinical Characterization of Emotional Dysregulation in Adults with and Without ADHD: A Cross-Sectional Multigroup Comparative Study

**DOI:** 10.3390/brainsci16040426

**Published:** 2026-04-18

**Authors:** Giulio Emilio Brancati, Elena Costagli, Alessandro Froli, Samuele Gemignani, Anna Magnesa, Ginevra Palazzesi, Pierpaolo Medda, Elisa Schiavi, Giulio Perugi

**Affiliations:** Psychiatry Unit 2, Department of Clinical and Experimental Medicine, University Hospital of Pisa, 56100 Pisa, Italy

**Keywords:** emotional dysregulation, attention-deficit/hyperactivity disorder, transdiagnostic psychopathology, psychiatric comorbidity, neurodevelopmental disorders, mood disorders

## Abstract

**Highlights:**

**What are the main findings?**

• Participants with ADHD + ED showed ADHD severity and executive dysfunction comparable to those with “pure” ADHD, while also exhibiting negative emotionality and affective comorbidity similar to those with “pure” ED.
• ADHD + ED was specifically associated with increased developmental comorbidity, earlier onset and greater cyclicity of comorbid mood disorders, and more pronounced and widespread functional impairments.

**What are the implications of the main findings?**

• ADHD + ED is unlikely to represent a more severe form of ADHD; rather, it may be clinically conceptualized as either a distinct entity or a comorbid phenotype, in which the complex interplay between ADHD and ED gives rise to distinct clinical features.
• Childhood emotional symptoms, early onset, neurodevelopmental comorbidity, and a family history of neurodevelopmental disorders support the conceptualization of ED as a neurodevelopmental condition.

**Abstract:**

**Background**: The association between emotional dysregulation (ED) and attention-deficit/hyperactivity disorder (ADHD) has been widely documented. However, a consensus has yet to be reached on how to conceptualize this domain within ADHD. Particularly, ADHD + ED may represent a distinct condition, a more severe ADHD, or a comorbidity. We explored these three main hypotheses, investigating clinical differences between patients with ADHD, ADHD + ED, and ED. **Methods**: In total, 101 participants (ages 18–50) were recruited and divided into four groups: ADHD (N = 23), ED (N = 28), ADHD + ED (N = 27), and HC (N = 23). ADHD and ED were assessed using the Diagnostic Interview for ADHD in adults (DIVA-5) and the Wender–Reimherr Adult Attention Deficit Disorder Scale (WRAADDS). Sociodemographic and clinical variables were compared among the groups. **Results**: Participants with ADHD and ADHD + ED exhibited comparably high ADHD severity and executive dysfunction ratings. While participants with ADHD + ED shared elevated negative emotionality and higher rates of mood disorders and anxiety disorders with participants with ED compared with those with ADHD, they showed specifically increased developmental and disruptive comorbidities, as well as earlier onset and greater cyclicity of comorbid mood disorders. Psychosocial functional impairments were similarly elevated on average in ADHD + ED and ADHD, but ADHD + ED showed more pronounced and widespread deficits across multiple domains. **Conclusions**: ADHD + ED is unlikely to represent a more severe form of ADHD but may be more appropriately conceptualized as either a distinct entity or a “comorbid” phenotype. Comparisons with ED without ADHD highlighted clinical features specific to ADHD + ED, including a higher neurodevelopmental load, impulse-control disorders, and a poorer course of mood disorders.

## 1. Introduction

Emotional dysregulation (ED) can be defined as a failure to regulate emotions resulting in emotional experiences or expressions that interfere with appropriate goal-directed activity [1,2]. While the term implies an impaired regulatory process, no consensus has been reached on the precise pathogenic mechanisms underlying ED, with some evidence pointing to abnormalities in stimulus processing and emotion generation, rather than to impaired regulation per se [3]. Although some authors have addressed these issues by adopting a narrower conceptualization of ED focused primarily on the inadequate use of regulatory strategies [4], accumulating evidence suggests that both top-down regulatory deficits and bottom-up abnormalities in emotion generation contribute to ED, with their relative contribution varying across clinical populations [5,6]. Accordingly, these frameworks are better viewed as complementary rather than mutually exclusive. In clinical practice, however, the label is commonly used to denote a clinical phenotype variably characterized by observable problematic behaviors or self-reported distressing emotional experiences, regardless of the underlying neurocognitive mechanisms [3].

Attention-deficit/hyperactivity disorder (ADHD) is a neurodevelopmental disorder characterized by developmentally inappropriate levels of inattention, hyperactivity, and impulsivity. While mostly diagnosed in children and adolescents, ADHD persistence into adulthood has been increasingly recognized in recent decades [7]. Although ED was originally recognized as a core symptom of ADHD in its early conceptualizations [8,9], current diagnostic criteria place greater emphasis on cognitive and behavioral dimensions of the disorder, most evident in the developmental age. However, mood instability, irritability, and emotional hyper-reactivity also represent common features in people diagnosed with ADHD [6,10,11].

Symptoms of ED have been observed in approximately 40% of children and 35–70% of adults with ADHD [12]. In children and adolescents, ED often manifests as abrupt and intense shifts in emotions (i.e., emotional lability), poor frustration tolerance, irritability, crying spells or tantrums, as well as overexpression of positive emotions, including exuberance and excitability [13,14]. In adults with ADHD, ED commonly presents as difficulties with emotional reactivity, mood swings, stress intolerance, anger outbursts, and interpersonal conflicts. Chronic mood instability has been reported in up to 90% of adults with ADHD in clinical samples [15].

Three main theoretical perspectives have emerged in the literature to account for the relationship between ADHD and ED, representing the most influential and empirically tested models [12]. According to some authors, the combination of ADHD and ED may define a distinct entity or ADHD subtype, characterized by specific clinical features, course, and distinct genetic bases [16,17]. Alternatively, ED may be considered a core feature of ADHD, integral to its psychopathology and related to its severity [11,18]. Finally, several authors view ED and ADHD as highly correlated but distinct dimensions, showing similar yet dissociable clinical courses and only partially sharing the same etiology [5]. In this latter perspective, ED is increasingly viewed as a transdiagnostic psychopathological feature potentially underlying several psychiatric conditions and their mutual comorbidity [2,19,20,21,22]. Accordingly, it may serve either as a specifier indicating risk for other comorbid disorders or as a separate “comorbid” condition co-occurring with ADHD. These perspectives are not mutually exclusive. Since ED may also be conceived as a heterogeneous phenomenon, including within ADHD [3], the different perspectives may apply to distinct clinical subgroups or to varying levels of severity.

These three theoretical models entail distinct therapeutic implications, although empirical evidence supporting specific treatment approaches for ED in ADHD remains limited. Should the “distinct entity” model be confirmed, further investigations aimed at developing specific interventions targeting patients with co-occurring ADHD and ED would be warranted [23]. Under the “severity” hypothesis, optimization of existing ADHD treatments—such as dose optimization or medication selection—would be required to ensure adequate management of ED symptoms. Indeed, there is evidence that standard ADHD pharmacotherapies may reduce ED, although findings are not always consistent [24]. Finally, if the “comorbidity” hypothesis is supported, combination or add-on treatment strategies addressing both conditions should be developed. Emerging research has begun to explore such approaches, including pharmacological interventions targeting emotion regulation specifically, but further studies are needed to establish their efficacy [25,26,27].

Several studies have compared patients with and without ED within ADHD samples [17,28,29,30,31]. These investigations consistently report a high prevalence of ED in both youths and adults with ADHD, as well as its association with symptom severity and functional impairment. While these observations may support the “severity” hypothesis, the frequent exclusion of participants with psychiatric comorbidities, together with the lack of systematic assessment of such comorbid conditions and the absence of clinical comparisons involving individuals presenting similar levels of ED in the absence of ADHD, limits the possibility of directly testing all hypotheses concurrently. Specifically, testing whether ED constitutes a distinct dimension co-occurring with ADHD requires comparing not only ADHD subgroups but also individuals presenting with clinically significant ED in the absence of ADHD, in order to evaluate whether the same correlates of ED are present irrespective of the ADHD diagnosis. Similarly, to test whether ADHD with ED represents a distinct subtype of ADHD, differences in symptom profile, comorbidity pattern, and clinical features should be observed relative to both ADHD alone and ED alone. Moreover, including an ED group without ADHD may permit examination of whether ED manifests similarly across diagnostic boundaries, consistent with its conceptualization as a transdiagnostic feature.

In this study, we aimed to examine clinical differences among patients with ADHD alone, patients with ADHD and co-occurring ED (ADHD + ED), patients with ED without ADHD, and HC. Although ED is conceptually a dimensional construct, a categorical operationalization was adopted to align with clinical reasoning, facilitate comparability with previous studies, and address the study’s primary aim. Consistent with the clinical framework outlined above, ED was operationalized as a phenotype characterized by emotion-related problematic behaviors and experiences—namely, mood swings, anger-related behavioral dyscontrol, and stress intolerance—without presupposing a specific underlying pathogenic mechanism. Group assignment was based on the Wender–Reimherr Adult Attention Deficit Disorder Scale (WRAADDS) using previously validated criteria in adult ADHD [17,28,32].

In line with the transdiagnostic conceptualization of ED, we included a group of patients with ED without ADHD, allowing us to examine whether the clinical correlates of ED are consistent across different diagnostic contexts or specific to ADHD. In contrast to previous studies that frequently excluded participants with psychiatric comorbidities, we adopted an inclusive approach to reflect real-world clinical populations, where the majority of adults with ADHD present with comorbid conditions. Comorbidity patterns were systematically assessed and compared across groups as part of our characterization of clinical differences.

The objective of this study was to evaluate evidence supporting or refuting the previously outlined theoretical models. According to the “distinct entity” hypothesis, ADHD + ED would be expected to show a distinct symptom profile, comorbidity pattern, clinical course, and psychosocial impairments compared with both “pure” ADHD and ED groups. In contrast, the “severity” hypothesis predicts that ADHD + ED would not differ qualitatively from the “pure” ADHD group but rather would show quantitative increases in symptom severity and functional impairment. Finally, the “comorbidity” hypothesis suggests that ADHD + ED would present features partly overlapping with ADHD (e.g., ADHD severity) on the one hand, and with ED (e.g., comorbidity) on the other, ultimately producing additive effects on clinical course and psychosocial impairment.

## 2. Materials and Methods

### 2.1. Recruitment

This monocentric, observational, cross-sectional, multigroup, case–control study was conducted between May and October 2024. Participants comprised patients clinically referred to the general or ADHD outpatient service of a tertiary-level psychiatry unit at the local university hospital, as well as self-referred individuals recruited through advertisements and snowball sampling. To be considered for participation, the following inclusion criteria were applied:

1. Clinical participants:
  - aged between 18 and 50 years;
  - capable of providing written informed consent;
  - capable of completing the clinical assessment;
  - satisfying criteria for one of the following conditions:
    (a) a current diagnosis of ADHD (ADHD group);
    (b) currently exhibiting significant ED symptoms (ED group);
    (c) presence of both inclusion criteria (a) and (b) (ADHD + ED);
2. HC:
  - aged between 18 and 50 years;
  - capable of providing written informed consent;
  - capable of completing the clinical assessment.

The following criteria were exclusionary:

1. Clinical participants:
  - remitted ADHD or ED or uncertain case status;
  - a history of psychosis or current psychotic symptoms;
  - current major affective episodes;
  - current alcohol or substance use disorder, excluding tobacco;
  - full-scale IQ (FSIQ) or general ability index (GAI) < 70;
  - history of brain injury or neurological conditions affecting cognition;
  - current treatment with methylphenidate, amphetamines, or atomoxetine.
2. HC:
  - criteria for inclusion in any clinical group;
  - evidence or suspicion of ADHD or ED in remission;
  - any current or past major psychiatric condition, except for provisional or persistent tic disorders, simple phobias, adjustment disorders, other specified eating disorders, and tobacco use disorder;
  - current or past non-suicidal self-injury (NSSI);
  - any current or past psychopharmacological treatment.

Psychopharmacological treatments other than methylphenidate, amphetamines, or atomoxetine were allowed in clinical groups, provided they had been administered for at least two months. Participants receiving ADHD medications were excluded, as the study included neuropsychological assessments whose findings will be presented in subsequent publications. Sustained remission of alcohol or substance use disorder, defined as a period lasting ≥12 months without meeting DSM-5 criteria other than craving, was permitted in clinical participants.

The sample size was determined a priori based on a balanced one-way analysis of variance (ANOVA) power calculation. In order not to miss clinically meaningful, large differences between groups (Cohen’s f = 0.4), with four groups planned (k = 4), a desired power of 0.9 was set, with a significance level of 0.05. Based on these parameters, the estimated number of participants per group was *n* ≈ 23, yielding a total sample size of at least 92 participants. Due to the potentially different prevalence or likelihood of recruitment of the conditions under study, consecutive recruitment continued until at least 23 participants had been enrolled in each group.

### 2.2. Clinical Assessment

All participants were evaluated by a single licensed psychiatrist in a single interview. For each patient, sociodemographic and clinical anamnestic data (e.g., major psychiatric conditions, family history, and psychopharmacological treatments) were recorded. All psychiatric conditions were diagnosed according to DSM-5 criteria, except for cyclothymic disorder, where both DSM-5 criteria and modified criteria were applied. Modified criteria included only criteria A-B and D-G from DSM-5, while the exclusion criterion C was modified, allowing a lifetime history of major depressive or hypomanic episodes. An early onset of cyclothymia (age ≤ 21 years), in accordance with Akiskal and Mallya criteria, was required to exclude patients with iatrogenic or residual mood swings. Autism spectrum traits were also recorded when persistent deficits in social communication and interaction, along with restricted, repetitive patterns of behavior, were present, even when a formal diagnosis of autism spectrum disorder could not be established, given uncertainty regarding the presence and level of impairment specifically attributable to autistic symptoms beyond other psychopathology and their course across life.

Criteria for ADHD based on DSM-5 were assessed using the Italian version of the Diagnostic Interview for ADHD in adults (DIVA-5), a semi-structured, clinician-administered interview, assessing ADHD symptoms in childhood and adulthood [33]. The Italian version of the WRAADDS was used to assess ADHD and ED symptom severity and is provided in the Supplementary Materials [34]. Specifically, the WRAADDS investigates seven target symptom domains in the Utah Criteria for adult ADHD, namely, (1) attention difficulties, (2) hyperactivity/restlessness, (3) temper, (4) affective lability, (5) emotional over-reactivity, (6) disorganization, and (7) impulsivity. Scores ranging from 0 = “none” to 4 = “very much” are assigned to each domain. In accordance with previous studies [17,28,32], significant ED symptoms were considered present whenever a participant scored ≥3 in at least two among domains 3 (temper), 4 (affective lability), and 5 (emotional over-reactivity). The history of ED symptoms during childhood (<12 years) and adolescence (12–18 years) was also collected. Significant ED symptoms were considered present in developmental ages whenever symptoms from two different domains were reported.

Both the WRAADDS and the DIVA-5 were administered, whenever possible, in the presence of an informant. Informant-reported questionnaires were considered to complement the clinical interview, particularly when the history of childhood symptoms was unclear. Informant-reported questionnaires were available for all but one participant, ensuring consistency in the availability of collateral information across groups.

### 2.3. Psychometric Assessment

Additional informant-reported and self-reported scales were used to assess ADHD symptom severity. Informant-reported scales included the Conners’ Adult ADHD Rating Scale—Observer: Screening Version (CAARS-O:SV) [35] and the Barkley Adult ADHD Rating Scale-IV—Other-report: childhood symptoms (BAARS-IV OR:CS) [36]. The CAARS-O:SV encompasses DSM-IV ADHD symptoms and the ADHD index, including items related to accessory features. The BAARS-IV explores DSM-IV childhood ADHD criteria, rated based on their frequency between 5 and 12 years, with scores from 1 = “never or rarely” to 4 = “very often”. In addition to continuous scores, BAARS-IV symptom counts were computed based on symptoms being present “often” or “very often” during childhood. Regarding self-reported questionnaires, current ADHD symptom severity was measured using the Adult ADHD Self-Report Scale—Version 1.1 (ASRS) [37], while childhood symptoms were investigated through the Wender Utah Rating Scale (WURS) [38].

Self-report and informant-report scales were also used to assess psychopathological and accessory features. The Behavior Rating Inventory of Executive Function (BRIEF-A) in its informant-report form [39] and the self-reported Adult Executive Functioning Inventory (ADEXI) [40] were used to measure executive dysfunctions. Self-reported symptoms of emotional dysregulation were assessed using the Reactivity, Intensity, Polarity, and Stability questionnaire in its 40-item version (RIPoSt-40) [41]. Affective temperamental traits were measured by means of the Temperament Evaluation of Memphis, Pisa, Paris, and San Diego—Münster version (TEMPS-M) [42,43]. Interpersonal sensitivity was evaluated through the Interpersonal Sensitivity Measure (IPSM) [44,45]. Impulsivity was assessed through two different self-report instruments, the short version of the Urgency, Perseverance, Premeditation, Sensation Seeking, and Positive Urgency Impulsive Behavior Scale (S-UPPS-P) [46] and the Barratt Impulsiveness Scale—15-item version (BIS-15) [47].

Finally, the Barkley Functional Impairment Scale—Full-length: Observer-Report version (BFIS-FL:OR) was used to assess psychosocial functional impairments over the past six months. It consists of 15 items evaluating a broad range of functional areas, including work, social relationships, and daily life activities. Each item, referring to a different area, is rated on a 0–9 scale. Lower scores (0–4) indicate none to mild difficulties, whereas higher scores (5–9) indicate moderate to severe impairments [48]. Item 15, related to child-rearing, was excluded from the analyses due to the low proportion of participants with children.

### 2.4. Statistical Analysis

All the statistical analyses were performed using R Statistical Software Version 4.3.1 (Foundation for Statistical Computing, Vienna, Austria) between February and August 2025.

Descriptive statistics were used to summarize the demographic and clinical characteristics of the groups. Sociodemographic variables, symptom severity, and functional impairments were compared among all groups. Clinical features, including psychiatric comorbidity, family history, and illness course, were compared among clinical groups. For all scales except the BFIS-FL:OR, missing items from subscales with less than 20% missing data were imputed using the respondent’s median of the completed items within the same subscale. Subscales with more than 20% missing data were considered invalid.

Subscale scores from the ADEXI, BIS-15, BRIEF-A, IPSM, RIPoSt-40, TEMPS-M, and S-UPPS-P were reduced into symptom dimensions using Principal Components Analysis (PCA). PCA was employed as an exploratory, data-driven approach to identify latent symptom dimensions without imposing a priori theoretical assumptions, while also reducing the number of variables to minimize multiple comparisons in subsequent analyses. Participants with 20% or more missing data from any subscale were excluded from the analysis. Adequacy of the data for PCA was confirmed by a Kaiser–Meyer–Olkin (KMO) measure of 0.84 and a significant Bartlett’s test of sphericity (*p* < 0.001). The number of components to retain was determined using Horn’s parallel analysis (paran package), which compares observed eigenvalues with those obtained from 990 randomly generated datasets. PCA was then performed using the psych package on the standardized correlation matrix, extracting three components as indicated by the parallel analysis and applying a varimax orthogonal rotation to facilitate interpretability. Components were interpreted based on factor loadings ≥0.4 and labeled according to the clinical constructs represented by the highest-loading subscales. Component scores were calculated using the regression method.

For continuous variables, comparative analyses were conducted using the Kruskal–Wallis test, followed by Dunn’s test for post hoc comparisons. One-way ANOVA with Dunn’s test was used to compare component scores. For categorical variables, Pearson’s chi-squared test (or Fisher’s exact test) was used, with pairwise Fisher’s exact tests used for post hoc analyses. False discovery rate correction was applied to post hoc contrasts to account for multiple comparisons. Effect sizes were calculated as standardized mean differences (SMD) and interpreted as small (≥0.2), medium (≥0.5), and large (≥0.8).

Exploratory multivariate linear regression models were conducted to examine the independent and combined effects of ADHD and ED as two distinct but potentially co-occurring conditions. Continuous outcomes (school years, PCA-derived dimensions, number of neurodevelopmental and psychiatric conditions, and BFIS-FL:OR average score) were regressed on ADHD and ED status, coded as categorical factors, along with their interaction term (ADHD × ED), and controlling for sex. The main effects of ADHD and ED were indicative of additive contributions, while the interaction term assessed potential synergistic effects, indicating whether the combined effect deviated from the sum of the individual effects. Due to sample size constraints and the binary nature of psychiatric comorbidity outcomes, interaction models could not be reliably estimated for binary variables.

A statistical significance threshold of *p* < 0.05 was set in all the analyses.

## 3. Results

### 3.1. Sample Characteristics

The sample was composed of 101 participants (44.4% males; age = 25.9 ± 5.5 years), including 23 diagnosed with ADHD, 27 with ADHD + ED, 28 with ED, and 23 HCs (Table 1). While 39 participants were clinically referred (N_ADHD_ = 12; N_ADHD+ED_ = 18; N_ED_ = 9), 62 self-referred participants were recruited through advertisements and snowball sampling (N_ADHD_ = 11; N_ADHD+ED_ = 9; N_ED_ = 19; N_HC_ = 23). Among them, 10 had been clinically followed up elsewhere (e.g., private practices and secondary-level services).

The ADHD group was composed of 16 participants with predominantly inattentive presentation, five with combined presentation, and two with predominantly hyperactive/impulsive presentation. The ADHD + ED group was composed of 17 participants with predominantly inattentive presentation and 10 with combined presentation.

Groups were comparable for age but not for sex. However, while a significant difference in sex distribution emerged among groups, no pairwise comparisons yielded statistically significant results after multiple comparison correction. An uncorrected significant difference was observed between ADHD and ED, with a male preponderance in the former and a female preponderance in the latter (*p* = 0.009, *p*_adj_ = 0.052).

As expected, significant differences were found for the educational level, with high school diploma and bachelor’s degree levels being significantly more common than master’s degree in the ADHD groups compared with HCs. A middle school diploma was also significantly more common than a master’s degree in ADHD + ED compared with HC. ADHD groups reported significantly fewer completed school years compared with HC. Moreover, participants with ADHD + ED had significantly fewer completed school years than participants with ED.

### 3.2. ADHD and ED Symptom Severity

As expected, significant differences in childhood and adulthood ADHD symptom severity were observed (Table 2). Across methods, ADHD and ADHD + ED exhibited similarly higher levels of core ADHD symptom severity compared with the other groups, with no significant differences observed for inattention and hyperactivity/impulsivity measures between the two. The only exception was for observer-reported inattentive symptoms on the CAARS:O-SV, where the ADHD + ED group scored significantly higher than “pure” ADHD.

The ED group scored significantly lower than the ADHD groups but higher than HC on overall current and childhood ADHD severity based on clinician-rated and self-reported scales. Particularly, self-reported current symptom severity on the ASRS-v1.1 did not differ significantly between ED and ADHD, though a significantly lower score was found in ED compared with ADHD + ED. Similarly, CAARS:O-SV ADHD index showed similar scores in ED and ADHD, which were significantly lower compared with ADHD + ED and higher compared with HC. Conversely, current observer-reported overall severity according to CAARS:O-SV showed no significant differences between ED and HC. In addition, while observer-rated childhood symptom severity on the BAARS-IV was significantly lower in the ED group than in the ADHD groups and higher than in HCs, BAARS-IV total symptom count showed no significant differences between the ED and HC.

With respect to ADHD symptom domains, participants with ED did not differ from HCs on DSM-based childhood and adulthood hyperactivity/impulsivity measures. Conversely, they had significantly higher scores on the WRAADDS impulsivity domain and the hyperactivity/impulsivity composite score. As for inattention, patients with ED scored significantly lower than ADHD groups and higher than HCs on current inattentive symptoms. While the same pattern was found for childhood inattention severity according to BAARS-IV, childhood inattentive symptom counts were not different between ED and HC.

As per the definition, the WRAADDS ED composite score, as well as the affective lability and emotional over-reactivity domains, was significantly higher in ED groups compared with ADHD and HC. “Pure” ADHD also scored significantly higher than HC on the composite ED score but not on the former individual domains. In contrast, the temper domain was significantly higher in all clinical groups compared with HC.

Self-reported childhood emotional symptoms on the WURS showed higher anxiety/dysphoria in all clinical groups compared with HC, and higher disruptive mood/behavior in the clinical groups compared with HC and in ADHD + ED compared with ED.

### 3.3. Other Symptom Dimensions

Symptom dimensions were derived through PCA of subscale scores from self-report and informant-report scales completed. In total, 91 participants were included in the analysis following imputation of missing values. Based on Horn’s parallel analysis, the adjusted eigenvalues for the first three components (11.91, 2.70, and 1.67) exceeded 1, indicating that three components, which together explained 60% of the total variance, should be retained. Based on the pattern of loadings, Component 1 was interpreted as negative emotionality, Component 2 as executive dysfunction, and Component 3 as encompassing both “pure” and emotion-related impulsivity (Table 3).

Participants with ADHD + ED and ED showed significantly greater negative emotionality compared with those with ADHD and HCs. In contrast, participants with ADHD and ADHD + ED demonstrated significantly more pronounced executive dysfunction compared with those with ED and HCs. Finally, all clinical groups showed significantly higher impulsivity scores compared with HC (Table 4).

### 3.4. Psychiatric Comorbidity and Clinical Features

Psychiatric comorbidity and clinical course were compared across clinical groups, excluding HC (Table 5). A gradient emerged, with the highest overall burden of psychiatric comorbidity observed in the ADHD + ED group, followed by the ED group, and then ADHD. This pattern was consistent when considering non-neurodevelopmental comorbidities, while neurodevelopmental comorbidities were significantly more frequent in ADHD + ED, with no significant difference between ED and ADHD.

Neurodevelopmental disorders, particularly autism spectrum conditions, as well as disruptive disorders, were primarily associated with ADHD + ED. In contrast, affective and anxiety disorders were significantly more common in both ADHD + ED and ED compared with ADHD. In particular, bipolar spectrum disorders, multiple anxiety disorders, panic attacks, and generalized anxiety were overrepresented in ED-related groups. Conversely, separation anxiety disorder and social anxiety disorder were more specifically linked to ADHD + ED.

The family history of bipolar disorder was significantly more common in participants from both ADHD groups than in participants with ED. Conversely, family history of anxiety disorder was more than twice as frequent in both ED groups than in those with ADHD, although differences were not significant at post hoc comparisons.

The prevalence of ED symptoms in childhood or adolescence was significantly higher in ADHD + ED than in the other groups. ED showed no significant differences for ED symptoms during childhood compared with the other groups, but during adolescence, it exhibited a significantly lower rate of ED symptoms compared with ADHD + ED and a higher rate compared with ADHD.

Among patients with mood disorders, ADHD + ED was associated with a lower age at onset of first major affective episode compared with ED, and at first depressive episode compared with both ADHD and ED. In addition, multiple affective episodes and multiple depressive episodes were significantly more common than no episodes in ADHD + ED compared with ADHD.

### 3.5. Psychosocial Functioning

Significant differences among groups were found for all areas of functioning except for romantic and/or sexual activities, which was the least endorsed item (Table 6).

Participants with ADHD + ED exhibited the greatest impairments across all areas, showing significantly more difficulties than all other groups in household chores, interactions with friends, and activities in the community. They also displayed significantly greater impairments in other social interactions, driving a motor vehicle, daily responsibilities, caring for oneself, and health maintenance compared with those with ED and HCs, but not with those with ADHD. Only participants with ADHD + ED had significantly greater impairments in work or occupational activities, intimate relationships, and money management compared with HCs.

Both ADHD and ADHD + ED demonstrated significantly greater difficulties than HC in educational activities, daily responsibilities, and caring for oneself. Differently, those with ADHD, as well as HCs, had significantly fewer problems in their home life with immediate family compared with those with ADHD + ED. No significant differences were found between the ED and HC groups.

Based on average BFIS-FL:OR scores, participants with ADHD and ADHD + ED demonstrated significantly greater functional impairments compared with HCs. In addition, those with ADHD + ED exhibited more severe difficulties than those with ED.

### 3.6. Interaction Analyses

Exploratory linear regression analyses were conducted to test the independent contributions of ADHD and ED, including their interaction and controlling for sex, for a subset of continuous outcomes (Table 7). The analyses confirmed a major association of ADHD with executive dysfunction and impaired psychosocial functioning based on informant report. ED was specifically related to negative emotionality. Additive effects of ADHD and ED were observed for years of education and impulsivity, with a greater impact of ADHD, as well as for the number of neurodevelopmental and psychiatric conditions, with a greater impact of ED. The effect of sex was significant only for negative emotionality, with higher scores in females.

## 4. Discussion

In this study, we examined clinical differences between patients with ADHD alone, ADHD + ED, ED without ADHD, and HCs. Sex distribution differed across groups, with a trend toward a higher proportion of males in the ADHD group and females in the ED group. Participants with ADHD + ED and “pure” ADHD exhibited reduced educational attainment and comparable levels of core ADHD symptom severity, except for informant-reported inattentive symptoms. ADHD + ED shared elevated negative emotionality and high rates of mood and anxiety disorder comorbidities with “pure” ED. Nevertheless, it was characterized by higher rates of neurodevelopmental and impulse-control disorders, as well as anxiety disorders, whose onset typically occurs during developmental age, namely, separation and social anxiety. In addition, ADHD + ED was associated with earlier onset and greater cyclicity of mood disorders, and more pronounced, pervasive psychosocial functional deficits across multiple domains compared with both “pure” ADHD and ED. Overall, our findings challenge the predictions of the “severity” model, while remaining compatible with both the “distinct entity” and the “comorbidity” hypotheses on the co-occurrence of ADHD and ED.

First, the gradient of sex distribution contrasted with the “severity” hypothesis, according to which a similar male predominance should have been observed in ADHD and ADHD + ED. Conversely, the intermediate male-to-female ratio observed in ADHD + ED may reflect, according to the “comorbidity” model, the additive effects of increased risk for ADHD in males and increased risk for ED in females; however, it is also compatible with the “distinct entity” hypothesis.

With respect to ADHD severity, most of the measures employed in this study indicated similar levels of core ADHD symptoms across the ADHD groups. This pattern contrasts with prior studies that have reported greater ADHD severity among individuals with ED in clinical populations [17,28,31,49,50,51]. Some studies may be partly biased by ED assessment instruments that incorporate attention-related symptoms [50] or focus predominantly on irritability and frustration sensitivity [49], as well as by analyses correlating ED and ADHD symptoms in samples including only patients with ADHD and HC, without other clinical populations [51]. Another source of inconsistency may also stem from the inclusion of non-clinically referred participants in our sample. Specifically, the association between ED and ADHD may be stronger in clinical settings but attenuated in community-based samples, as indicated by community studies showing less robust links between emotional dimensions and ADHD symptoms relative to those observed within core ADHD domains or with related traits such as impulsivity [52,53]. Collectively, these findings support only a partial overlap between ADHD and ED.

Likewise, the dissociation between symptom dimensions derived from self-report and informant-report scales provides empirical support for the distinction between ED and ADHD as separable yet interacting dimensions. Specifically, negative emotionality was elevated in both ED groups, executive dysfunction in both ADHD groups, and impulsivity in all clinical groups compared to HC. While some measures shared item content with the clinician-rated WRAADDS used for group classification, the convergence between independent sources supports the validity of the clinical distinctions made in this study. Moreover, the specific elevation of the executive dysfunction dimension in ADHD groups—with similar scores for both patients with and without ED—argues against the interpretation of ED as simply reflecting general psychopathology severity. If this were the case, an increased severity of executive dysfunction would have been expected in the ADHD + ED group. Conversely, the finding that impulsivity was elevated across all clinical groups, with no significant differences among clinical groups, suggests that impulsivity represents a nonspecific transdiagnostic feature rather than a distinguishing characteristic of ADHD or ED.

Comorbidity rates were largely consistent with previous research comparing patients with ADHD with and without ED. The elevated prevalence of mood, anxiety, and impulse-control comorbidities observed in our ADHD + ED group mirrors prior findings linking ED to greater psychiatric comorbidity in ADHD populations [29,49,50,54,55,56,57,58,59], as well as longitudinal investigations suggesting that ED may act as a precursor to subsequent psychiatric comorbidity in ADHD [60,61,62,63]. The finding of more pronounced and pervasive functional impairments among participants with ADHD + ED also aligns with prior studies demonstrating that ED contributes to functional impairment beyond the effects of inattentive and hyperactive/impulsive symptoms, in both children and adults [64,65,66,67,68].

However, to the best of our knowledge, our study is the first to compare patients with and without ADHD who have similar levels of ED, allowing us to determine which features of patients with ADHD + ED are specific to ADHD and which are common to other conditions associated with ED. Compared with “pure” ED, ADHD + ED showed an increased rate of neurodevelopmental disorders, particularly autism spectrum conditions, and impulse-control comorbidities. Considering the well-documented co-occurrence of ADHD and other neurodevelopmental disorders or disruptive comorbidities [69,70,71,72,73], it is unsurprising that participants with ADHD + ED exhibit higher rates than those with ED alone. However, the selective increase in patients with ADHD with ED—absent in participants without ADHD—suggests a distinctive triple association among ED, ADHD, and these comorbidities, in line with prior research.

In previous studies, autism spectrum disorders—with or without co-occurring ADHD—and ADHD with comorbid oppositional defiant disorders have been associated with a higher prevalence of ED compared with ADHD alone [74,75,76]. Significant associations between autism spectrum traits and both ED and comorbid psychopathology have been reported in children and adults with ADHD [58,77,78,79,80], and the presence of ADHD in children with ASD has been linked to increased severity of comorbid psychopathology, especially in the domain of externalizing symptoms [81,82,83,84]. Similarly, greater psychopathology severity and ED have been associated with comorbid oppositional defiant disorder among patients with ADHD [17,59,76,80,85], and some evidence also indicates increased ED in those with oppositional defiant disorder co-occurring with ADHD relative to those without ADHD [86]. In addition, neurodevelopmental comorbidity has been linked to both ED and disruptive behavior disorders in youths with mood disorders [87], and one study reported that the co-occurrence of ADHD with both autism spectrum and disruptive behavior disorders was specifically associated with ED, underlining a complex interplay between these conditions [80].

Patients with ADHD + ED also specifically showed earlier onset and greater cyclicity of mood disorders compared with those with “pure” ED. These observations are consistent with studies reporting earlier onset, a higher number of episodes, and greater impairment in patients with major mood disorders comorbid with ADHD relative to non-comorbid individuals [88,89,90,91,92]. However, this is the first study, to our knowledge, showing that these effects are mainly attributable to the co-occurrence of ADHD and ED, rather than being associated with ADHD per se.

While the similar affective comorbidity observed in ADHD + ED and ED aligns with the “comorbidity” model, the prominence of developmental comorbidities and the poorer course of mood disorders in ADHD + ED seem to be better understood within the framework of the “distinct entity” hypothesis. However, the specifically elevated rates of disruptive disorders, separation anxiety, and social anxiety, along with the poorer course of mood disorders, may also reflect—consistent with the “comorbidity” hypothesis—potential synergistic effects of ADHD and ED, in which the interaction of two putatively distinct processes produces emergent phenomena not predicted by either condition alone. For instance, a reduced threshold for emotion generation underlying ED could lead to disruptive behaviors only when combined with the impaired inhibitory control associated with ADHD. Similarly, ADHD-related erratic behaviors, such as substance abuse or circadian rhythm dysregulation, may trigger earlier mood episodes in susceptible individuals and exacerbate cyclicity.

On the other hand, the co-occurrence of ADHD + ED and autism spectrum traits may either indicate a distinct clinical entity or reflect a multiplicative effect of the neurodevelopmental burden associated with both ADHD and ED. Indeed, in our sample, a neurodevelopmental component of ED was suggested by retrospective reports of childhood emotional symptoms, a high prevalence of early-onset ED, neurodevelopmental comorbidity rates comparable to those observed in “pure” ADHD, and similar rates of family history of neurodevelopmental disorders across all clinical groups. Future studies are warranted to further explore neurodevelopmental correlates and origins of ED.

Although recognizing ED as a neurodevelopmental condition may aid in the interpretation of our findings, it remains plausible that the diverse clinical phenotypes encompassed by the ADHD + ED designation arise from heterogeneous mechanisms, including those attributable to comorbidity between ADHD and ED and others requiring distinct underlying processes. However, consistent with this principle of parsimony, the present study focused on testing more straightforward hypotheses based on comparisons across clinically defined groups. Empirically examining heterogeneity within the ADHD + ED group (e.g., distinguishing between synergistic comorbidity versus a distinct entity) was therefore beyond the scope of the present study and would have required larger sample sizes. Notably, the “comorbidity” hypothesis—which posits that ADHD and ED are distinct dimensions whose co-occurrence may produce synergistic effects—offers a more parsimonious framework than the “distinct entity” hypothesis, as it does not require postulating a separate underlying condition. Importantly, larger samples are also required to empirically test the distinction between the “comorbidity” and “distinct entity” hypotheses through formal interaction analyses. Under the “comorbidity” hypothesis, ADHD and ED would show either no interaction (i.e., only additive effects) or a positive synergistic interaction, meaning that their combined effect is greater than the sum of their individual effects. Under the “distinct entity” hypothesis, a negative interaction would be expected—that is, the combined condition would deviate from the pattern predicted by the additive effects of ADHD and ED alone, suggesting a qualitatively separate entity. Our preliminary interaction analyses on a small subset of continuous variables appear to be more consistent with the former hypothesis. However, given the binary nature of comorbidity outcomes and the strong overlap observed in our study between some comorbid conditions and ADHD + ED and/or ED, further studies with larger samples are needed to reliably estimate interaction effects within this fundamental domain.

### Limitations

Several methodological constraints should be acknowledged. First, the study was monocentric. While this design ensured consistent recruitment and assessment procedures, it may limit external validity due to potential influences of local diagnostic practices and healthcare systems. Specifically, this study was conducted in a single Italian clinical setting, and findings may not be directly generalizable to other healthcare systems with different diagnostic practices, referral pathways, and cultural attitudes toward mental health. Replication in diverse cultural and clinical contexts and in multicenter samples is warranted to confirm the generalizability of our findings.

All clinical assessments were performed by a single clinician. While this ensured consistency in diagnostic procedures, it precluded the calculation of inter-rater reliability. However, in a previous study from our group, excellent inter-rater reliability (Cohen’s k = 0.82) was demonstrated for the assessment of ED using overlapping criteria [93].

A mixed recruitment strategy was adopted, including both clinically referred patients and self-referred participants recruited through advertisements and snowball sampling. While this approach likely reduced the risk of oversampling patients with more severe conditions typical of tertiary care settings, it also limits comparability with purely clinical samples and may have introduced selection bias, as self-referred participants may differ from clinically referred individuals in terms of help-seeking behavior, symptom awareness, and illness severity.

The cross-sectional nature of this study also restricts the possibility of improving diagnostic accuracy, evaluating clinical course, and drawing causal inferences about observed associations. In addition, several exclusion criteria were applied, which may limit generalizability.

Although the sample size was determined a priori to ensure a 90% power to detect large, clinically meaningful differences between groups (Cohen’s f = 0.4), more subtle effects—potentially relevant for theoretical understanding—may have been overlooked. Null findings for smaller effect sizes should be interpreted with caution, as they may reflect insufficient statistical power rather than the true absence of group differences. In addition, the present study was not powered to examine age- or sex-related contributions to the differences observed across groups, and stratified analyses would have been underpowered given the sample size in each group. Although the four groups were comparable in terms of age, the sex distribution showed a trend-level difference between the ADHD and ED groups, which did not survive correction for multiple comparisons. Future studies with larger or more balanced samples should systematically investigate age and sex differences among groups and their potential moderating effects. On the other hand, omnibus tests were not corrected for multiple comparisons, which may have increased the risk of false positives; replication in independent samples is therefore warranted.

Treatment response was not explored, although this could represent a relevant avenue for testing models of the co-occurrence of ADHD and ED and for disentangling the heterogeneity of ED. Some participants were receiving psychopharmacological treatment at the time of assessment. Although ADHD-specific treatment and recent medication changes were exclusion criteria, residual treatment effects on current symptomatology cannot be completely ruled out. A systematic exploration of treatment effects was beyond the primary aims of this study and would have required subgroup analyses for which the sample size was insufficient, given treatment heterogeneity.

A further limitation of this study is the lack of etiological investigation of ADHD and ED, including genetic, familial, and environmental factors. Disentangling the contribution of these factors is not feasible on a purely clinical basis without the systematic assessment of family members and comprehensive genetic analyses. The heterogeneity within the ADHD + ED group was also not empirically examined. In line with the principle of parsimony, we prioritized testing the simpler hypotheses. Examining distinct subtypes or heterogeneous pathways to ED would have required larger sample sizes and was beyond the scope of the present study. Future research integrating genetic, familial, and environmental assessments is needed to better clarify the multifactorial etiology of ADHD and ED, as well as their co-occurrence.

While many psychopathological dimensions were investigated through multiple raters, functional impairment was assessed only based on informant report, which may introduce bias. Future studies should incorporate multiple informants (e.g., self-report and clinician-rated) and objective indicators to provide a more comprehensive assessment of functional outcomes.

Finally, the definition of ED employed in this study also presents some limitations. To enhance reproducibility and minimize subjective biases, it was entirely based on clinical manifestations, and regulatory mechanisms were neither included in the definition nor assessed. Furthermore, although ED can be conceptualized dimensionally, a categorical approach was adopted here to better align with clinical reasoning and the study’s primary aim of comparing clinically defined groups. Nevertheless, we acknowledge that a dimensional operationalization may capture additional variance in ED not reflected in categorical group assignment. Although group classification and outcome measures were derived from different instruments and raters, we cannot completely exclude the possibility of shared method variance or that the definition of ED groups may have influenced the observed findings.

In addition, although the WRAADDS has been established as a commonly used assessment instrument for ED in individuals with ADHD, several more granular facets of ED may not be fully captured by its scores. For instance, aspects such as frustration intolerance, maladaptive attempts to suppress negative emotional states, impulsive reactions to positive emotions, withdrawal or inaction in response to sadness, and the generalization of negative self- or worldviews are not directly captured by this tool. Additionally, specific features of mood fluctuations—such as their duration and whether they are spontaneous or reactive, or congruent or incongruent with stimuli—are also overlooked. These facets may provide valuable insights for a better clinical characterization of the heterogeneous population with ED-related problems in future studies.

## 5. Conclusions

The distinct clinical characteristics of patients with ADHD + ED, relative to both “pure” ADHD and ED, suggest that ADHD + ED may not be adequately captured as merely a more severe form of ADHD. Rather, it may be more appropriately conceptualized as either a distinct entity or subtype of ADHD, as a “comorbid” phenotype with ED conferring a higher risk for affective comorbidity, or as a heterogeneous group encompassing patients for whom both hypotheses apply. Certain clinical features, such as a higher prevalence of impulse-control disorders and a more adverse course of mood disorders, are compatible with both the “distinct entity” hypothesis and the synergistic effects hypothesized under the “comorbidity” framework. By contrast, the elevated rate of neurodevelopmental comorbidities appears to be a specific characteristic that cannot be fully explained by additive or synergistic mechanisms, and may instead reflect greater neurodevelopmental alterations underlying the co-occurrence of ADHD and ED.

## Figures and Tables

**Table 1:** Sociodemographic characteristics of the sample stratified by clinical condition.

	ADHD (N = 23)	ADHD + ED (N = 27)	ED (N = 28)	HC (N = 23)				
	*M* *[IQR]* *N (%)*	*M [IQR] * *N (%)*	*M [IQR] * *N (%)*	*M [IQR] * *N (%)*	*χ* ^2^	*SMD*	*p*	*Post hoc*
**Sex (male)**	14 (60.9)	12 (44.4)	6 (21.4)	11 (47.8)	8.57	0.437	0.036	ns
**Age (years)**	25.00 [21.00, 27.50]	25.00 [21.50, 28.00]	25.50 [23.75, 28.50]	26.00 [22.50, 28.00]		0.216	0.799	
**Marital status**						0.500	0.715	
Single	11 (47.8)	11 (40.7)	11 (39.3)	11 (47.8)				
Stable relationship	9 (39.1)	12 (44.4)	12 (42.9)	11 (47.8)				
Cohabiting	1 (4.3)	4 (14.8)	2 (7.1)	0 (0.0)				
Married	2 (8.7)	0 (0.0)	2 (7.1)	1 (4.3)				
Separated	0 (0.0)	0 (0.0)	1 (3.6)	0 (0.0)				
**Occupational status**						0.677	0.425	
Unemployed	1 (4.3)	4 (14.8)	3 (10.7)	2 (8.7)				
Employed full-time	5 (21.7)	6 (22.2)	7 (25.0)	3 (13.0)				
Employed part-time	1 (4.3)	5 (18.5)	2 (7.1)	0 (0.0)				
Self-employed	1 (4.3)	2 (7.4)	1 (3.6)	2 (8.7)				
Student	12 (52.2)	9 (33.3)	13 (46.4)	16 (69.6)				
Working student	3 (13.0)	1 (3.7)	2 (7.1)	0 (0.0)				
**Educational level**						0.923	0.003	HC > ADHD ≃ ADHD + ED
Middle school	2 (8.7)	3 (11.1) c	1 (3.6)	0 (0.0)				
High school	15 (65.2) a	19 (70.4) d	14 (50.0)	10 (43.5) ad				
Bachelor’s degree	4 (17.4) b	4 (14.8) e	4 (14.3)	1 (4.3) be				
Master’s degree	0 (0.0) ab	1 (3.7) cde	7 (25.0)	10 (43.5) abcde				
Postgraduate	2 (8.7)	0 (0.0)	2 (7.1)	2 (8.7)				
**School years**	14.00 [13.00, 15.50]	13.00 [13.00, 15.00]	16.00 [14.00, 18.00]	18.00 [16.50, 19.00]		0.853	<0.001	HC > ADHD ≃ADHD + ED; ED > ADHD + ED
**Repeated grades**	3 (13.0)	5 (18.5)	2 (7.1)	0 (0.0)		0.384	0.135	

Abbreviations: ADHD = attention-deficit/hyperactivity disorder; ED = emotional dysregulation; HC = healthy controls; IQR = interquartile range; M = median; ns = not significant; SMD = standardized mean difference.

**Table 2:** ADHD scores stratified by clinical condition.

	ADHD (N = 23)	ADHD + ED (N = 27)	ED (N = 28)	HC (N = 23)			
DIVA-5 Adult Symptom Criteria	M [IQR] m (SD)	M [IQR] m (SD)	M [IQR] m (SD)	M [IQR] m (SD)	SMD	*p*	Post Hoc
Inattention	6.00 [5.00, 7.50] 6.22 (1.76)	8.00 [6.00, 9.00] 7.33 (1.57)	4.00 [2.00, 6.00] 4.14 (2.69)	0.00 [0.00, 1.50] 0.91 (1.47)	2.001	<0.001	ADHD ≃ ADHD + ED > ED > HC
Hyperactivity/impulsivity	3.00 [1.50, 5.00] 3.09 (2.02)	4.00 [2.50, 5.00] 3.96 (2.01)	1.00 [0.00, 2.00] 1.39 (1.55)	0.00 [0.00, 1.00] 0.48 (0.85)	1.248	<0.001	ADHD ≃ ADHD + ED > ED ≃ HC
Total symptom criteria	10.00 [7.00, 10.50] 9.30 (2.67)	11.00 [10.00, 12.50] 11.30 (2.80)	5.50 [4.00, 7.00] 5.54 (3.50)	1.00 [0.00, 2.50] 1.39 (1.67)	2.186	<0.001	ADHD ≃ ADHD + ED > ED > HC
**DIVA-5 child symptom criteria**							
Inattention	4.00 [3.00, 5.00] 4.39 (1.88)	4.00 [3.00, 7.00] 5.15 (2.23)	1.00 [0.00, 2.00] 1.14 (1.21)	0.00 [0.00, 0.00] 0.17 (0.49)	1.977	<0.001	ADHD ≃ ADHD + ED > ED ≃ HC
Hyperactivity/impulsivity	3.00 [2.00, 5.50] 3.48 (2.56)	4.00 [2.00, 6.00] 4.07 (2.40)	1.00 [0.00, 1.00] 0.93 (1.12)	0.00 [0.00, 0.50] 0.39 (0.72)	1.250	<0.001	ADHD ≃ ADHD + ED > ED ≃ HC
Total symptom criteria	8.00 [4.00, 10.00] 7.87 (3.77)	9.00 [6.00, 12.00] 9.22 (3.83)	2.00 [1.00, 3.00] 2.07 (1.54)	0.00 [0.00, 0.50] 0.57 (1.08)	1.945	<0.001	ADHD ≃ ADHD + ED > ED > HC
**WRAADDS domain scores**							
1. Attention difficulties	3.00 [2.00, 3.00] 2.52 (0.67)	3.00 [2.50, 3.00] 2.89 (0.64)	2.00 [1.00, 2.00] 1.82 (1.09)	0.00 [0.00, 1.00] 0.48 (0.67)	1.797	<0.001	ADHD ≃ ADHD + ED > ED > HC
2. Hyperactivity/restlessness	2.00 [1.50, 2.00] 1.83 (0.94)	2.00 [1.50, 2.00] 1.93 (0.78)	1.00 [0.00, 1.00] 0.82 (0.77)	0.00 [0.00, 1.00] 0.48 (0.59)	1.169	<0.001	ADHD ≃ ADHD + ED > ED ≃ HC
3. Temper	1.00 [0.00, 2.00] 1.00 (0.95)	1.00 [1.00, 3.00] 1.63 (1.08)	1.00 [1.00, 2.00] 1.29 (0.94)	0.00 [0.00, 0.00] 0.17 (0.49)	0.929	<0.001	ADHD ≃ ADHD + ED ≃ ED > HC
4. Affective lability	2.00 [1.00, 2.00] 1.78 (0.85)	3.00 [3.00, 4.00] 3.37 (0.49)	3.00 [3.00, 3.00] 3.11 (0.74)	1.00 [0.00, 1.00] 0.83 (0.89)	1.969	<0.001	ADHD + ED ≃ ED > ADHD ≃ HC
5. Emotional over-reactivity	2.00 [0.50, 2.00] 1.43 (1.08)	3.00 [3.00, 3.50] 3.07 (0.78)	3.00 [3.00, 3.00] 3.04 (0.33)	1.00 [0.00, 1.00] 0.87 (0.69)	1.899	<0.001	ADHD + ED ≃ ED > ADHD ≃ HC
6. Disorganization	3.00 [2.00, 3.00] 2.78 (0.74)	3.00 [3.00, 4.00] 3.22 (0.70)	2.00 [1.00, 2.00] 1.96 (1.00)	0.00 [0.00, 1.00] 0.48 (0.67)	2.011	<0.001	ADHD ≃ ADHD + ED > ED > HC
7. Impulsivity	2.00 [1.00, 2.00] 1.52 (0.99)	2.00 [1.00, 3.00] 1.89 (0.89)	1.00 [0.00, 2.00] 1.14 (1.04)	0.00 [0.00, 1.00] 0.35 (0.49)	1.025	<0.001	ADHD > HC; ADHD + ED > ED > HC
**WRAADDS composite scores**							
Total domains score	13.00 [10.50, 14.50] 12.87 (3.27)	18.00 [16.00, 19.00] 18.00 (2.80)	13.50 [11.00, 15.00] 13.18 (3.42)	4.00 [1.00, 6.00] 3.65 (2.60)	2.480	<0.001	ADHD + ED > ADHD ≃ ED > HC
Inattention/disorganization	5.00 [5.00, 6.00] 5.30 (1.06)	6.00 [5.00, 6.50] 6.11 (1.01)	4.00 [2.00, 5.00] 3.79 (1.95)	1.00 [0.00, 2.00] 0.96 (1.07)	2.348	<0.001	ADHD ≃ ADHD + ED > ED > HC
Hyperactivity/impulsivity	3.00 [2.00, 4.00] 3.35 (1.67)	4.00 [3.00, 5.00] 3.81 (1.36)	2.00 [1.00, 3.00] 1.96 (1.50)	1.00 [0.00, 1.00] 0.83 (0.89)	1.314	<0.001	ADHD ≃ ADHD + ED > ED > HC
Combined ADHD	8.00 [7.00, 9.50] 8.65 (2.17)	9.00 [8.50, 11.50] 9.93 (1.90)	6.00 [4.00, 7.25] 5.75 (2.94)	1.00 [0.00, 3.00] 1.78 (1.65)	2.208	<0.001	ADHD ≃ ADHD + ED > ED > HC
Emotional dysregulation	4.00 [3.00, 5.50] 4.22 (1.83)	8.00 [7.00, 9.00] 8.07 (1.36)	7.00 [7.00, 8.00] 7.43 (0.96)	2.00 [1.00, 3.00] 1.87 (1.42)	2.601	<0.001	ADHD + ED ≃ ED > ADHD > HC
**CAARS:O-SV**							
Inattentive Symptoms	12.00 [5.00, 15.50] 11.48 (6.34)	16.00 [13.50, 17.50] 15.59 (4.89)	6.00 [3.00, 11.00] 7.11 (5.11)	2.00 [1.00, 5.00] 3.78 (4.07)	1.329	<0.001	ADHD + ED > ADHD > ED > HC
Hyperactive/Impulsive Symptoms	8.00 [5.00, 11.00] 8.39 (4.98)	9.00 [5.00, 16.00] 10.52 (6.66)	3.50 [2.75, 6.25] 4.18 (2.89)	1.00 [0.00, 3.00] 2.65 (4.34)	0.945	<0.001	ADHD ≃ ADHD + ED > ED ≃ HC
ADHD Symptoms Total	20.00 [12.00, 25.00] 19.87 (9.17)	25.00 [21.00, 31.00] 26.11 (8.94)	11.00 [5.75, 15.25] 11.29 (6.94)	4.00 [2.00, 8.50] 6.43 (7.34)	1.383	<0.001	ADHD ≃ ADHD + ED > ED ≃ HC
ADHD Index	16.00 [10.00, 19.50] 15.30 (6.87)	21.00 [15.00, 26.50] 20.93 (6.38)	10.50 [8.00, 15.25] 11.43 (5.24)	2.00 [2.00, 8.00] 5.48 (5.86)	1.373	<0.001	ADHD + ED > ADHD ≃ ED > HC
**BAARS-IV OR:CS scores**							
Total score §	39.00 [27.00, 43.50] 36.28 (9.35)	40.00 [31.25, 49.75] 41.62 (10.80)	25.00 [22.00, 28.00] 25.21 (4.87)	20.50 [19.00, 23.00] 20.77 (2.35)	1.679	<0.001	ADHD ≃ ADHD + ED > ED > HC
Inattention score §	18.00 [14.25, 24.50] 19.50 (6.09)	23.00 [19.25, 29.75] 23.69 (6.42)	12.50 [11.00, 15.25] 13.32 (3.37)	10.50 [9.00, 12.00] 10.73 (1.83)	1.600	<0.001	ADHD ≃ ADHD + ED > ED > HC
Hyperactivity/Impulsivity score *	16.00 [12.50, 21.00] 16.78 (5.06)	17.00 [12.50, 22.75] 17.92 (6.17)	11.00 [9.75, 13.25] 11.89 (3.07)	10.00 [9.00, 11.00] 10.30 (1.87)	1.100	<0.001	ADHD ≃ ADHD + ED > ED ≃ HC
**BAARS-IV OR:CS symptom counts**							
Total §	6.00 [1.50, 9.00] 5.83 (4.15)	8.00 [3.25, 10.00] 7.54 (4.44)	0.00 [0.00, 2.00] 1.11 (1.50)	0.00 [0.00, 0.00] 0.00 (0.00)	1.547	<0.001	ADHD ≃ ADHD + ED > ED ≃ HC
Inattention §	2.00 [1.00, 5.00] 3.22 (2.76)	5.00 [2.25, 7.00] 4.92 (2.86)	0.00 [0.00, 0.00] 0.54 (1.10)	0.00 [0.00, 0.00] 0.00 (0.00)	1.447	<0.001	ADHD ≃ ADHD + ED > ED ≃ HC
Hyperactivity/Impulsivity *	2.00 [0.50, 4.00] 2.61 (2.31)	2.00 [1.00, 4.00] 2.62 (2.32)	0.00 [0.00, 1.00] 0.57 (1.03)	0.00 [0.00, 0.00] 0.09 (0.42)	0.989	<0.001	ADHD ≃ ADHD + ED > ED ≃ HC
**ASRS-v1.1**							
Total score	15.00 [13.00, 16.00] 14.17 (2.48)	16.00 [15.00, 17.50] 15.78 (2.56)	13.00 [11.00, 15.00] 12.68 (2.54)	5.00 [2.50, 8.00] 5.52 (4.22)	1.657	<0.001	ADHD ≃ ED > HC; ADHD + ED > ED > HC
**WURS**							
Total score *	41.00 [27.75, 52.50] 39.32 (14.11)	50.00 [38.50, 58.00] 47.35 (14.93)	29.00 [16.75, 36.25] 27.84 (12.97)	9.00 [4.50, 17.00] 11.35 (7.96)	1.630	<0.001	ADHD ≃ ADHD + ED > ED > HC
Disruptive mood/behavior *	13.50 [9.00, 19.75] 13.82 (7.06)	17.00 [11.75, 23.50] 17.65 (8.71)	9.00 [4.00, 13.25] 9.62 (7.00)	4.00 [1.50, 6.50] 4.43 (3.50)	1.118	<0.001	ADHD ≃ ED > HC; ADHD + ED > ED > HC
ADHD *	18.00 [12.25, 22.00] 16.91 (6.24)	21.00 [14.50, 24.50] 19.41 (6.55)	11.00 [6.00, 14.25] 10.39 (5.17)	2.00 [1.00, 4.50] 3.00 (2.68)	1.838	<0.001	ADHD ≃ ADHD + ED > ED > HC
Anxiety/dysphoria *	6.50 [4.25, 9.75] 6.73 (3.72)	8.00 [6.50, 11.00] 8.70 (3.70)	6.00 [3.00, 10.00] 6.68 (4.19)	3.00 [1.00, 5.00] 3.35 (2.87)	0.770	<0.001	ADHD ≃ ADHD + ED ≃ ED > HC

Abbreviations: ADHD = attention-deficit/hyperactivity disorder; ASRS-v1.1 = Adult ADHD Self-Report Scale—Version 1.1; BAARS-IV OR:CS = Barkley Adult ADHD Rating Scale-IV Other report: Childhood Symptoms; CAARS:O-SV = Conners’ Adult ADHD Rating Scale—Observer: Screening Version; DIVA-5 = Diagnostic Interview for ADHD in Adults; ED = emotional dysregulation; HC = healthy controls; IQR = interquartile range; M = median; m = mean; SD = standard deviation; SMD = standardized mean difference; WRAADDS = Wender–Reimherr Adult Attention Deficit Disorder Rating Scale; WURS = Wender Utah Rating Scale. § N = 99; * N = 100.

**Table 3:** Symptom dimensions according to Principal Component Analysis (PCA) with varimax rotation.

Subscale	Component 1 *Negative* *Emotionality*	Component 2 *Executive* *Dysfunction*	Component 3 *Impulsivity*	u^2^	Complexity
RIPoSt-40—Negative emotionality	**0.85**	0.20	0.23	0.18	1.3
IPSM—Separation anxiety	**0.83**	0.19	0.16	0.25	1.2
TEMPS-M—Depressive temperament	**0.81**	0.15	0.07	0.32	1.1
IPSM—Interpersonal awareness	**0.78**	0.13	0.01	0.38	1.1
IPSM—Fragile inner-self	**0.75**	0.04	0.22	0.38	1.2
TEMPS-M—Anxious temperament	**0.70**	0.20	0.14	0.45	1.2
RIPoSt-40—Affective instability	**0.69**	0.19	**0.46**	0.28	1.9
RIPoSt-40—Emotional impulsivity	**0.66**	0.19	**0.54**	0.23	2.1
TEMPS-M—Cyclothymic temperament	**0.64**	0.10	**0.51**	0.31	2.0
BRIEF-A—Shift	**0.62**	**0.46**	−0.04	0.40	1.9
BRIEF-A—Emotional control	**0.52**	0.25	0.07	0.66	1.5
TEMPS-M—Hyperthymic temperament	**−0.50**	−0.13	**0.46**	0.52	2.1
IPSM—Need for approval	**0.40**	0.24	0.05	0.78	1.7
IPSM—Timidity	0.38	0.18	−0.09	0.81	1.5
BRIEF-A—Plan/Organize	0.15	**0.86**	0.23	0.19	1.2
BRIEF-A—Task monitor	0.21	**0.83**	0.14	0.25	1.2
BRIEF-A—Working memory	0.30	**0.82**	0.19	0.20	1.4
BRIEF-A—Initiate	0.34	**0.81**	0.10	0.22	1.4
BRIEF-A—Inhibit	0.14	**0.80**	0.22	0.30	1.2
BRIEF-A—Organization of materials	0.26	**0.77**	0.18	0.30	1.3
BRIEF-A—Self-monitor	0.18	**0.56**	0.30	0.56	1.7
BIS-15—Lack of attention	0.39	**0.54**	**0.46**	0.35	2.8
ADEXI—Working memory	0.39	**0.51**	**0.49**	0.35	2.9
BIS-15—Pure impulsivity	0.13	0.32	**0.78**	0.28	1.4
S-UPPS-P—Positive urgency	0.15	0.09	**0.75**	0.41	1.1
S-UPPS-P—Negative urgency	0.31	0.04	**0.68**	0.43	1.4
ADEXI—Inhibition	0.24	0.47	**0.67**	0.27	2.1
S-UPPS-P—Lack of premeditation	−0.09	0.34	**0.65**	0.46	1.6
S-UPPS-P—Sensation seeking	−0.23	0.03	**0.64**	0.53	1.3
TEMPS-M—Irritable temperament	0.29	0.10	**0.60**	0.55	1.5
BIS-15—Planning difficulties	−0.04	0.44	**0.58**	0.47	1.9
BIS-15—Impulsive buying	0.17	0.30	**0.49**	0.64	1.9
S-UPPS-P—Lack of perseverance	0.34	0.41	**0.48**	0.49	2.8
**Eigenvalue**	7.44	6.39	5.97		
**Proportion of variance explained**	0.23	0.19	0.18		
**Cumulative variance explained**	0.23	0.42	0.60		

Loadings ≥ 0.4 are shown in bold. Abbreviations: ADEXI = Adult Executive Functioning Inventory; BIS-15 = Barratt Impulsiveness Scale—15-item version; BRIEF-A = Behavior Rating Inventory of Executive Function—Adult version; IPSM = Interpersonal Sensitivity Measure; RIPoSt-40 = Reactivity, Intensity, Polarity and Stability questionnaire—40-item version; TEMPS-M = Temperament Evaluation of Memphis, Pisa, Paris, and San Diego—Münster; S-UPPS-P = Short Urgency, Perseverance, Premeditation, Sensation Seeking, and Positive Urgency Impulsive Behavior Scale.

**Table 4:** Symptom dimensions stratified by clinical condition.

	ADHD (N = 20)	ADHD + ED (N = 24)	ED (N = 24)	HC (N = 23)				
Component	m (SD)	m (SD)	m (SD)	m (SD)	F	SMD	*p*	Post Hoc
1. Negative emotionality	−0.41 (0.97)	0.61 (0.85)	0.48 (0.77)	−0.78 (0.67)	16.02	1.053	<0.001	ADHD + ED ≃ ED > ADHD ≃ HC
2. Executive dysfunction	0.58 (0.97)	0.48 (0.76)	−0.35 (1.09)	−0.63 (0.56)	10.71	0.898	<0.001	ADHD + ED ≃ ADHD > ED ≃ HC
3. Impulsivity	0.32 (0.83)	0.47 (1.08)	−0.11 (0.93)	−0.66 (0.76)	6.98	0.714	<0.001	ADHD + ED ≃ ADHD ≃ ED > HC

Abbreviations: ADHD = attention-deficit/hyperactivity disorder; ED = emotional dysregulation; HC = healthy controls; m = mean; SD = standard deviation; SMD = standardized mean difference.

**Table 5:** Clinical differences between participants diagnosed with ADHD alone, with ADHD and ED, and with ED alone.

	ADHD (N = 23)	ADHD + ED (N = 27)	ED (N = 28)				
Psychiatric Comorbidities	*M* *[IQR]* *N (%)*	*M [IQR] * *N (%)*	*M [IQR] * *N (%)*	*χ* ^2^	*SMD*	*p*	*Post Hoc*
Number of neurodevelopmental and psychiatric conditions	2.00 [0.50, 4.00]	6.00 [4.00, 7.50]	4.00 [3.00, 5.00]		1.077	<0.001	ADHD + ED > ED > ADHD
Number of neurodevelopmental conditions	0.00 [0.00, 0.00]	1.00 [0.00, 1.00]	0.00 [0.00, 0.00]		0.549	0.002	ADHD + ED > ADHD ≃ ED
Number of psychiatric conditions	2.00 [0.00, 3.50]	5.00 [4.00, 7.00]	3.00 [2.00, 5.00]		0.994	<0.001	ADHD + ED > ED > ADHD
**Neurodevelopmental disorders comorbidity**							
Autism spectrum conditions					0.709	0.008	ns
Autism spectrum disorder	1 (4.3)	6 (22.2)	1 (3.6)				
Autism spectrum traits	4 (17.4)	4 (14.8)	0 (0.0)				
No autism spectrum traits	18 (78.3)	17 (63.0)	27 (96.4)				
Autism spectrum disorder or traits	5 (21.7)	10 (37.0)	1 (3.6)		0.608	0.006	ADHD + ED > ED
Other neurodevelopmental disorders	4 (17.4)	12 (44.4)	5 (17.9)	6.45	0.408	0.040	ns
**Mood disorders comorbidity**							
Any mood disorder	11 (47.8)	26 (96.3)	23 (82.1)	17.11	0.841	<0.001	ADHD + ED ≃ ED > ADHD
Mood disorder type					1.084	<0.001	ADHD + ED ≃ ED > ADHD
Bipolar and related disorders	5 (21.7) ^abc^	22 (81.5) ^b^	20 (71.4) ^ac^				
Depressive disorders	6 (26.1) ^c^	4 (14.8)	3 (10.7) ^c^				
No mood disorders	12 (52.2) ^ab^	1 (3.7) ^b^	5 (17.9) ^a^				
Mood disorder diagnosis					1.350	<0.001	ADHD + ED ≃ ED > ADHD
Other specified bipolar disorder	2 (8.7)	2 (7.4)	1 (3.6)				
Bipolar disorder type 2 with cyclothymia	1 (4.3) ^a^	8 (29.6) ^a^	5 (17.9)				
Major depressive disorder with cyclothymia	1 (4.3)	10 (37.0)	10 (35.7)				
Cyclothymic disorder	1 (4.3)	2 (7.4)	4 (14.3)				
Major depressive disorder (recurrent)	0 (0.0)	0 (0.0)	1 (3.6)				
Major depressive disorder (single episode)	4 (17.4)	3 (11.1)	1 (3.6)				
Persistent depressive disorder	2 (8.7)	1 (3.7)	1 (3.6)				
No mood disorders	12 (52.2) ^a^	1 (3.7) ^a^	5 (17.9)				
**Anxiety disorders comorbidity**							
Any anxiety disorder	8 (34.8)	20 (74.1)	23 (82.1)	13.89	0.717	0.001	ADHD + ED ≃ ED > ADHD
Multiple anxiety disorders	3 (13.0)	14 (51.9)	12 (42.9)	8.61	0.599	0.013	ADHD + ED ≃ ED > ADHD
Separation anxiety disorder	0 (0.0)	13 (48.1)	6 (21.4)	15.83	0.895	<0.001	ADHD + ED > ED > ADHD
Social anxiety disorder	7 (30.4)	19 (70.4)	12 (42.9)	8.53	0.570	0.014	ADHD + ED > ADHD
Panic disorder	2 (8.7)	7 (25.9)	4 (14.3)		0.312	0.280	
Panic attack specifier	1 (4.3)	11 (40.7)	8 (28.6)	8.82	0.639	0.012	ADHD + ED ≃ ED > ADHD
Generalized anxiety disorder	3 (13.0)	13 (48.1)	17 (60.7)	12.33	0.738	0.002	ADHD + ED ≃ ED > ADHD
**Disruptive and impulse-control disorders comorbidity**							
Any disruptive, impulse-control, and conduct disorder	1 (4.3)	8 (29.6)	0 (0.0)		0.645	<0.001	ADHD + ED > ED ≃ ADHD
Oppositional defiant disorder	1 (4.3)	6 (22.2)	0 (0.0)		0.534	0.006	ADHD + ED > ED
Intermittent explosive disorder	0 (0.0)	5 (18.5)	0 (0.0)		0.449	0.005	ns
**Other psychiatric comorbidities**							
Any obsessive-compulsive or related disorder	6 (26.1)	15 (55.6)	11 (39.3)	4.51	0.414	0.105	
Any trauma- and stressor-related disorder	0 (0.0)	3 (11.1)	2 (7.1)		0.343	0.312	
Any somatic symptom and related disorder	2 (8.7)	4 (14.8)	3 (10.7)		0.127	0.824	
Any eating disorder	9 (39.1)	17 (63.0)	13 (46.4)	3.05	0.325	0.218	
Any substance-related or addictive disorder	4 (17.4)	6 (22.2)	7 (25.0)	0.43	0.125	0.805	
**First-degree family history**							
Neurodevelopmental disorders	4 (17.4)	5 (18.5)	5 (17.9)		0.020	1.000	
Attention-deficit/hyperactivity disorders	4 (17.4)	4 (14.8)	3 (10.7)		0.129	0.850	
Mood disorders	11 (47.8)	19 (70.4)	14 (50.0)	3.30	0.313	0.192	
Bipolar or related disorders	5 (21.7)	6 (22.2)	0 (0.0)		0.504	0.013	ADHD ≃ ADHD + ED > ED
Depressive disorders	7 (30.4)	16 (59.3)	14 (50.0)	4.25	0.400	0.119	
Anxiety disorders	4 (17.4)	13 (48.1)	13 (46.4)	6.14	0.461	0.047	-
Obsessive-compulsive and related disorders	1 (4.3)	2 (7.4)	1 (3.6)		0.113	0.835	
Eating disorders	3 (13.0)	6 (22.2)	4 (14.3)		0.162	0.693	
Substance use disorders	4 (17.4)	2 (7.4)	3 (10.7)		0.205	0.610	
Psychosis	1 (4.3)	1 (3.7)	1 (3.6)		0.027	1.000	
Suicide attempts	1 (4.3)	1 (3.7)	1 (3.6)		0.027	1.000	
**Clinical course and features**							
Significant ED symptoms during childhood	8 (34.8)	21 (77.8)	14 (50.0)	9.75	0.626	0.008	ADHD + ED > ADHD
Significant ED symptoms during adolescence	10 (43.5)	27 (100.0)	21 (75.0)	20.82	1.035	<0.001	ADHD + ED > ED > ADHD
Significant ED symptoms during childhood or adolescence	11 (47.8)	27 (100.0)	21 (75.0)	18.36	0.958	<0.001	ADHD + ED > ADHD ≃ ED
Consulted mental health professionals	19 (82.6)	26 (96.3)	25 (89.3)		0.308	0.299	
Currently receiving psychopharmacological treatment	3 (13.0)	11 (40.7)	11 (39.3)	5.43	0.438	0.066	
Age at first mental health consultation (N = 70)	18.00 [10.00, 22.00]	15.00 [12.00, 19.50]	18.00 [13.00, 23.00]		0.269	0.330	
Age at onset of mood disorders (N = 58)	17.00 [14.50, 21.00]	15.00 [13.00, 16.00]	14.50 [13.00, 17.00]		0.580	0.097	
Age at first major affective episode (N = 50)	17.50 [15.50, 23.75]	17.00 [16.00, 18.75]	20.00 [18.25, 22.75]		0.647	0.006	ED > ADHD + ED
Age at first major depressive episode (N = 46)	20.00 [17.00, 26.00]	17.00 [16.00, 18.25]	20.00 [18.00, 22.00]		0.812	0.003	ADHD ≃ ED > ADHD + ED
Age at first hypomanic episode (N = 15)	15.00 [14.50, 20.00]	17.00 [15.00, 19.00]	20.00 [19.00, 21.00]		0.726	0.222	
Major affective episodes				12.21	0.735	0.016	ADHD + ED > ADHD
No major episodes	14 (60.9) ^a^	6 (22.2) ^a^	11 (39.3)				
Single episode	6 (26.1)	5 (18.5)	7 (25.0)				
Multiple episode	3 (13.0) ^a^	16 (59.3) ^a^	10 (35.7)				
Major depressive episodes				11.71	0.729	0.020	ADHD + ED > ADHD
No major depressive episodes	15 (65.2) ^a^	7 (25.9) ^a^	12 (42.9)				
Single episode	6 (26.1)	6 (22.2)	7 (25.0)				
Multiple episode	2 (8.7) ^a^	14 (51.9) ^a^	9 (32.1)				
Hypomanic episodes					0.473	0.142	
No hypomanic episodes	20 (87.0)	20 (74.1)	23 (82.1)				
Single episode	2 (8.7)	1 (3.7)	4 (14.3)				
Multiple episode	1 (4.3)	6 (22.2)	1 (3.6)				
History of NSSI	3 (13.0)	9 (33.3)	10 (35.7)	3.74	0.364	0.154	
History of suicide attempts	0 (0.0)	5 (18.5)	2 (7.1)		0.471	0.084	

Abbreviations: ADHD = attention-deficit/hyperactivity disorder; ED = emotional dysregulation; IQR = Interquartile Range; M = median; ns = not significant; NSSI = non-suicidal self-injury; SMD = standardized mean difference.

**Table 6:** Psychosocial functioning impairments stratified by clinical condition.

		ADHD (N = 23)	ADHD + ED (N = 27)	ED (N = 28)	HC (N = 23)			
BFIS-FL:OR Domains	Missing (%)	M [IQR] m (SD)	M [IQR] m (SD)	M [IQR] m (SD)	M [IQR] m (SD)	SMD	*p*	Post Hoc
1. Home life with immediate family	8.9	1.00 [0.00, 3.50] 2.00 (2.16)	4.00 [1.75, 7.00] 4.04 (2.79)	2.00 [0.00, 3.25] 2.39 (2.39)	1.00 [0.00, 3.00] 1.38 (1.80)	0.591	0.005	ADHD + ED > ADHD ≃ HC
2. Managing the household/household chores	30.7	2.00 [0.00, 3.50] 2.27 (2.22)	5.00 [4.25, 7.00] 5.61 (1.88)	1.00 [0.00, 3.00] 1.65 (1.66)	0.00 [0.00, 1.25] 0.80 (1.32)	1.417	<0.001	ADHD + ED > ADHD ≃ ED ≃ HC
3. Work or occupational activities	11.9	3.00 [0.00, 5.00] 3.00 (2.94)	3.00 [1.50, 5.00] 3.30 (2.58)	1.00 [0.00, 2.00] 1.85 (2.44)	1.00 [0.00, 2.00] 0.95 (1.16)	0.612	0.010	ADHD + ED > HC
4. Social interactions with strangers or acquaintances	0.0	2.00 [0.00, 4.50] 2.43 (2.46)	4.00 [1.50, 5.00] 3.30 (2.22)	0.50 [0.00, 2.25] 1.54 (1.91)	0.00 [0.00, 1.00] 0.91 (1.62)	0.656	0.001	ADHD + ED > ED ≃ HC
5. Social interactions with friends	1.0	1.00 [0.00, 3.00] 1.57 (1.95)	3.00 [1.00, 5.00] 3.08 (2.45)	0.00 [0.00, 1.00] 0.82 (1.47)	0.00 [0.00, 1.00] 0.57 (0.90)	0.744	<0.001	ADHD + ED > ADHD ≃ ED ≃ HC
6. Activities in the community	31.7	0.00 [0.00, 2.00] 1.60 (2.59)	4.00 [2.50, 6.00] 4.20 (2.88)	0.00 [0.00, 1.00] 1.14 (1.86)	0.00 [0.00, 0.00] 0.47 (1.23)	0.847	0.001	ADHD + ED > ADHD ≃ ED ≃ HC
7. Educational activities	11.9	4.00 [1.00, 7.00] 4.10 (3.16)	3.50 [2.00, 8.00] 4.28 (3.27)	2.00 [0.00, 5.00] 2.74 (2.94)	0.00 [0.00, 1.00] 1.00 (1.71)	0.699	<0.001	ADHD + ED ≃ ADHD > HC
8. Intimate relationships	23.8	1.00 [0.00, 5.00] 2.76 (3.09)	3.50 [1.00, 6.00] 3.55 (2.77)	2.00 [0.00, 3.00] 2.38 (2.20)	0.00 [0.00, 1.00] 1.06 (1.71)	0.551	0.024	ADHD + ED > HC
9. Money management/financial affairs	4.0	1.00 [0.00, 5.50] 2.65 (3.24)	2.00 [1.00, 7.00] 3.58 (3.47)	0.00 [0.00, 2.75] 1.96 (2.89)	0.00 [0.00, 1.00] 1.14 (1.91)	0.464	0.032	ADHD + ED > HC
10. Driving a motor vehicle	24.8	1.00 [0.00, 2.00] 1.53 (1.81)	3.00 [0.00, 5.00] 3.00 (2.69)	0.00 [0.00, 2.00] 1.17 (1.95)	0.00 [0.00, 0.25] 1.15 (2.52)	0.418	0.024	ADHD + ED > ED ≃ HC
11. Romantic and/or sexual activities	35.6	1.00 [0.00, 5.00] 2.57 (2.87)	2.00 [0.00, 5.00] 2.93 (3.06)	1.00 [0.00, 5.00] 2.47 (2.79)	0.00 [0.00, 1.00] 0.95 (1.81)	0.405	0.075	
12. Organization and management of daily responsibilities	0.0	4.00 [1.00, 5.50] 3.78 (3.10)	5.00 [3.50, 7.00] 5.00 (2.60)	1.00 [0.00, 4.00] 2.07 (2.45)	0.00 [0.00, 1.00] 0.83 (1.56)	0.992	<0.001	ADHD > HC; ADHD + ED > ED ≃ HC
13. Caring for oneself	0.0	3.00 [0.50, 5.00] 3.26 (2.85)	4.00 [1.50, 6.00] 4.11 (3.06)	1.00 [0.00, 2.00] 1.79 (2.44)	0.00 [0.00, 1.50] 0.87 (1.39)	0.763	<0.001	ADHD > HC; ADHD + ED > ED ≃ HC
14. Health maintenance	0.0	2.00 [0.00, 5.00] 2.78 (2.76)	5.00 [3.00, 8.00] 5.22 (2.95)	2.00 [0.00, 3.25] 2.61 (2.70)	0.00 [0.00, 2.00] 1.26 (1.94)	0.773	<0.001	ADHD + ED > ED ≃ HC
**BFIS** **-FL:OR average score**	0.0	2.43 [0.70, 4.35] 2.76 (2.13)	3.82 [2.80, 4.58] 3.91 (1.64)	1.70 [0.45, 2.74] 1.86 (1.58)	0.36 [0.11, 1.62] 0.99 (1.16)	1.011	<0.001	ADHD > HC; ADHD + ED > ED ≃ HC

Abbreviations: ADHD = attention-deficit/hyperactivity disorder; BFIS-FL:OR = Barkley Functional Impairment Scale—Full-length: Observer-Report; ED = emotional dysregulation; HC = healthy controls; IQR = interquartile range; M = median; m = mean; SD = standard deviation; SMD = standardized mean difference.

**Table 7:** Linear regression models testing the independent and combined effects of ADHD and ED, controlling for sex.

	ADHD		ED		ADHD × ED		Sex (Female)	
Outcomes	Estimate (SE)	*p*	Estimate (SE)	*p*	Estimate (SE)	*p*	Estimate (SE)	*p*
**School years**	−3.16 (0.76)	<0.001	−1.87 (0.74)	0.013	1.04 (1.03)	0.315	0.47 (0.54)	0.394
**Component scores**								
Negative emotionality	0.46 (0.24)	0.056	1.14 (0.23)	<0.001	−0.20 (0.33)	0.548	0.56 (0.17)	0.002
Executive dysfunction	1.25 (0.27)	<0.001	0.22 (0.26)	0.394	−0.35 (0.36)	0.335	0.24 (0.19)	0.206
Impulsivity	0.95 (0.28)	0.001	0.58 (0.27)	0.034	−0.42 (0.39)	0.280	−0.14 (0.20)	0.488
**Neurodevelopmental and** **psychiatric conditions**	1.73 (0.60)	0.005	3.16 (0.58)	<0.001	0.45 (0.81)	0.581	0.62 (0.43)	0.147
**BFIS-FL:OR average score**	1.83 (0.49)	<0.001	0.76 (0.47)	0.113	0.32 (0.66)	0.635	0.43 (0.35)	0.219

Abbreviations: ADHD = attention-deficit/hyperactivity disorder; BFIS-FL:OR = Barkley Functional Impairment Scale—Full-length: Observer-Report; ED = emotional dysregulation; SE = standard error.

## Data Availability

The raw data supporting the conclusions of this article will be made available by the authors on request.

## Supplementary Materials

The following supporting information can be downloaded at: https://www.mdpi.com/article/10.3390/brainsci16040426/s1, File S1: Scala Wender-Reimherr per il Disturbo da Deficit di Attenzione nell’Adulto

## Abbreviations

The following abbreviations are used in this manuscript:

ADEXI	Adult Executive Functioning Inventory
ADHD	Attention-Deficit/Hyperactivity Disorder
ASRS	Adult ADHD Self-Report Scale—Version 1.1
BIS-15	Barratt Impulsiveness Scale—15-item version
BFIS-FL:OR	Barkley Functional Impairment Scale—Full-length: Observer-Report
BRIEF-A	Behavior Rating Inventory of Executive Function—Adult version
BAARS-IV OR:CS	Barkley Adult ADHD Rating Scale-IV—Other-report: Childhood Symptoms
CAARS-O:SV	Conners’ Adult ADHD Rating Scale—Observer: Screening Version
DIVA-5	Diagnostic Interview for ADHD in adults, Third Edition
DSM-IV	Diagnostic and Statistical Manual of Mental Disorders, Fourth Edition
DSM-5	Diagnostic and Statistical Manual of Mental Disorders, Fifth Edition
ED	Emotional Dysregulation
FSIQ	Full-Scale Intelligence Quotient
GAI	General Ability Index
HC	Healthy Controls
IPSM	Interpersonal Sensitivity Measure
PCA	Principal Component Analysis
RIPoSt-40	Reactivity, Intensity, Polarity, and Stability questionnaire—40-item version
S-UPPS-P	Urgency, Perseverance, Premeditation, Sensation Seeking, and Positive Urgency Impulsive Behavior Scale—Short version
TEMPS-M	Temperament Evaluation of Memphis, Pisa, Paris, and San Diego—Münster version
WRAADDS	Wender–Reimherr Adult Attention Deficit Disorder Scale
WURS	Wender Utah Rating Scale
