# Supplementary material for: Clinical Characterization of Emotional Dysregulation in Adults with and Without ADHD: A Cross-Sectional Multigroup Comparative Study
Source: Brain Sci. 2026 Apr 18;16(4):426. doi: 10.3390/brainsci16040426 (PMC13115240; doi:10.3390/brainsci16040426)
Supplement: Supplementary file 1 [file brainsci-16-00426-s001.zip › brainsci-4234319-supplementary.pdf]

Scala Wender-Reimherr per il Disturbo da Deficit di Attenzione nell'Adulto

Scala e guida all'intervista italiana

Questa intervista ha lo scopo di misurare la gravità dei sette sintomi inclusi nei Criteri dello Utah in adulti con sintomatologia correlata al Disturbo da Deficit di Attenzione e Iperattività (*Attention-Deficit/Hyperactivity Disorder*; *ADHD*). I sintomi devono essere presenti cronicamente, sebbene possano essere aggravati da stress. La valutazione viene effettuata al meglio se somministrata durante un'intervista congiunta con il soggetto e un informatore, quale il coniuge o un partner.

Le singole domande devono essere seguite da domande generali al fine di meglio comprendere l'estensione e la gravità di un problema particolare:

Quanto è grave questo problema?
Quanto spesso è accaduto?
Ci sono stati commenti da parte di altri?
Cosa hanno detto?
Che difficoltà o problemi ha causato con gli altri, sul lavoro o a scuola?

Ai singoli item deve essere attribuito un punteggio secondo la seguente scala:

0 – Per nulla, non presente
1 – Lieve, in parte o a volte vero
2 – Chiaramente presente o spesso vero

I punteggi riassuntivi devono basarsi sui punteggi attribuiti ai singoli item nonché su ogni altro sintomo dello stesso ambito riportato dal soggetto. Il punteggio riassuntivo non deve essere una semplice media dei singoli punteggi, ma deve includere piuttosto la valutazione dell'estensione e della gravità dei deficit all'interno del dominio. Per esempio, se un solo item viene valutato come chiaramente presente, un punteggio riassuntivo di "4" potrebbe essere appropriato se questo singolo fattore è causa di problemi significativi.

Punteggi riassuntivi:

0 – Assente
1 – Lieve
2 – Moderato
3 – Abbastanza elevato
4 – Molto elevato

1. Difficoltà attentive:
si manifestano con incapacità di rimanere concentrati sulle conversazioni, distraibilità (la tendenza a posare l'attenzione su altri stimoli nonostante i tentativi di filtrarli), difficoltà nel mantenere la concentrazione su materiali di lettura o attività, la mente spesso "da un'altra parte", "tra le nuvole".

1. Difficoltà di attenzione
Punteggio riassuntivo 0-4:
0 1 2 3 4

a. Ridotta durata attentiva
Fa fatica a concentrarsi su un singolo contenuto del pensiero per periodi ragionevoli di tempo

Hai difficoltà a mantenere l'attenzione, a concentrarti? La mente tende a vagare e andare dove vuole?
0 1 2

b. Distraibilità
Non è in grado di ritrovare la concentrazione se interrotto

Ti distrai facilmente? Fai fatica a concentrarti se ci sono suoni o altre distrazioni?
0 1 2

c. Difficoltà di ascolto
Spesso manca il punto delle discussioni

Trovi difficile rimanere concentrato sulle conversazioni? Gli altri si lamentano che non li ascolti, che non fai attenzione a loro mentre ti parlano?
0 1 2

d. Ascolto all'interno di un pubblico
(è possibile utilizzare programmi di informazione o educativi come ambito di indagine alternativo se il soggetto non partecipa alle attività elencate)

Nei contesti formali, come lezioni, riunioni, conferenze, trasmissioni, o in chiesa, fai fatica a prestare attenzione a chi parla? (Non attribuire un punteggio se il soggetto non partecipa a queste attività)
0 1 2

e. Attenzione durante la lettura

Fai fatica a mantenere la concentrazione mentre leggi? Eviti o non gradisci la lettura di nulla che non sia di particolare interesse per te? Devi spesso rileggere perché la tua mente va da un'altra parte o fai fatica a comprendere materiali scritti?
0 1 2

<u>**2. Iperattività motoria persistente:**</u><br>si manifesta con irrequietezza, incapacità di rilassarsi, "nervosismo" (col significato di incapacità di calmarsi – non di ansia anticipatoria), incapacità di perdurare in attività sedentarie (per es. guardare un film, la TV, leggere il giornale), tendenza a essere sempre in movimento, a essere insoddisfatto se inattivo.	**2. Iperattività/Irrequietezza**<br>**Punteggio riassuntivo 0-4:**<br>0 1 2 3 4
**a. Sentimenti di tensione, irrequietezza**<br>Sentimenti interiori, nervosismo	Hai difficoltà a rilassarti? Ti senti spesso irrequieto o teso?<br>0 1 2
**b. Iperattività, irrequietezza fisica**	Sei uno che non sta mai fermo? Senti il bisogno di essere sempre in movimento o indaffarato? Fai fatica a rimanere seduto a lavoro, a scuola o a casa? Devi spesso alzarti e muoverti? Sei incapace di rimanere seduto durante un film o un programma TV?<br>0 1 2
**c.** ***Fidgetiness*** [tendenza a produrre continuamente piccoli movimenti che spesso infastidiscono le persone intorno]**, incapacità di rimanere fermo**	Stai sempre a muovere le mani o i piedi? La gente lo nota? Fai fatica a rimanere fermo, cambi spesso posizione? Tamburelli con le dita, giochi con gli oggetti, muovi le gambe, ti mangi le unghie, ti arricci i capelli o sbatti i piedi? (valutare sia sulla base della risposta che dell'osservazione)<br>0 1 2

3. Temperamento irascibile, accessi di rabbia rapidi e improvvisi:
il soggetto riporta di avere transitorie perdite di controllo e di esserne spaventato dal proprio comportamento. È facilmente provocabile o costantemente irritabile. I problemi di gestione della rabbia interferiscono con le relazioni personali.

3. Rabbia
Punteggio riassuntivo 0-4:
0 1 2 3 4

a. Irritabilità

Ti senti spesso irritabile o arrabbiato nei confronti del coniuge, dei figli o di altri membri della famiglia, oppure a lavoro, alla guida, o in altre situazioni?
0 1 2

b. Scatti d'ira

Hai attacchi di rabbia o ti innervosisci facilmente? Vai facilmente in escandescenza o perdi facilmente le staffe?
0 1 2

c. Perdita di controllo
Interferenza nel lavoro o nelle relazioni personali

La tua irascibilità ti causa problemi? Perdi il controllo durante gli scatti d'ira? (dire cose di cui ti penti, diventare aggressivo, agire in maniera minacciosa o fare gesti impulsivi)
0 1 2

4. Labilità affettiva:
generalmente descritta come antecedente all'adolescenza e in alcuni casi presente fin dalla prima infanzia. Si manifesta con spiccate oscillazioni da un umore normale alla depressione o all'euforia lieve o – più spesso – all'eccitamento; la depressione è descritta come tendenza a sentirsi "giù", "annoiato" o "insoddisfatto"; solitamente le oscillazioni dell'umore durano da qualche ora ad al massimo pochi giorni e si presentano senza significative variazioni fisiologiche concomitanti; le oscillazioni dell'umore possono accadere spontaneamente o essere reattive.

4. Labilità affettiva
Punteggio riassuntivo 0-4:
0 1 2 3 4

Prima di attribuire un punteggio, l'intervistatore dovrebbe differenziare tra un disturbo dell'umore maggiore e la labilità dell'umore dei soggetti con *ADHD*. La disforia associata ad *ADHD* è generalmente breve, dura ore, e solitamente ha un evento scatenante identificabile. Fa eccezione il caso di soggetti che sperimentano persistenti problemi di vita (spesso auto-procuratisi), in cui la durata della disforia può essere estesa. Allo stesso modo, è necessario distinguere l'eccitamento (che può essere lieve) e l'entusiasmo dall'umore elevato di qualità maniacale.

I soggetti con *ADHD* possono presentare comorbidità con una depressione maggiore. È necessario determinare la durata e la frequenza degli episodi e la presenza di condizioni somatiche concomitanti per distinguere lo scoraggiamento, il carattere lunatico e la demoralizzazione che si osservano nell'*ADHD* dalla depressione maggiore caratterizzata da perdita di interesse e perdita di capacità di provare piacere.

a. Fluttuazioni dell'umore
(differiscono dall'iperattività dal momento che quest'ultima è cronica e persistente)

Il tuo umore cambia spesso, fa sue e giù – come una montagna russa, con alti e bassi continui?
0 1 2

b. Periodi disforici

Hai spesso periodi in cui ti senti triste, giù o scoraggiato? Durante questi periodi, sei estremamente auto-critico o ti butti giù?
0 1 2

c. Noia

Ti senti spesso annoiato? Perdi facilmente interesse nelle cose?
0 1 2

d. Iper-stimolazione
Diventare troppo eccitato, essere troppo rumoroso, esuberante. Può presentarsi in situazioni sociali

Hai periodi in cui sei eccessivamente attivo, su di giri, ti entusiasmi troppo, vai troppo veloce, o parli troppo?
0 1 2

**<u>5. Iper-reattività emotiva:</u>**<br>il soggetto non riesce a fronteggiare stress ordinari e reagisce eccessivamente o inappropriatamente con depressione, confusione, incertezza, ansia o rabbia. Le risposte emotive interferiscono con la capacità di risolvere i problemi in maniera appropriata. Il paziente sperimenta ripetute crisi nel gestire stress della vita di tutti i giorni. Si descrive come facilmente "sopraffatto" dai problemi o "esaurito".	**1. Iper-reattività emotiva**<br>**Punteggio riassuntivo 0-4:**<br>0 1 2 3 4
**a. Sopraffatto, emotivo**<br>Percepisce le cose intensamente	Hai spesso la sensazione di essere sopraffatto dai problemi? Ti senti spesso "sotto pressione" o frustrato?<br>0 1 2
**b. Reattività**	Reagisci in maniera eccessiva allo stress, tendi a preoccuparti in modo esagerato? Piccoli problemi ti sembrano troppo difficili, tendi a ingigantirli oltremisura?<br>0 1 2
**c. Compromissione**<br>Sotto stress anche semplici responsabilità sono troppo impegnative	Quando queste reazioni hanno luogo, fai fatica a gestire le tue attività o a completare i tuoi compiti? Sotto pressione o stress, diventi ansioso, disorganizzato o confuso?<br>0 1 2

6. Disorganizzazione, incapacità di portare a termine le attività:
il soggetto riporta mancanza di organizzazione nel lavoro, nelle faccende domestiche o nei compiti scolastici; le attività vengono spesso lasciate incompiute; il soggetto passa da un'attività all'altra in maniera disordinata; disorganizzazione nelle attività, nella risoluzione dei problemi, nell'organizzazione del tempo, mancanza di costanza, frequente smemoratezza; tendenza a perdere spesso o mettere fuori posto oggetti, dimenticare piani, chiavi della macchina, portafoglio, ecc.

6. Disorganizzazione
Punteggio riassuntivo 0-4:
0 1 2 3 4

a. Organizzazione in generale

Hai problemi con l'organizzazione a casa, a lavoro o a scuola? Fai fatica a organizzare il tuo tempo, a stabilire priorità, a lavorare in maniera ordinata?
0 1 2

b. Problemi nell'aderenza ai compiti
I compiti non vengono completati o le cose richiedono più tempo a causa della tendenza a saltare da un'attività a un'altra

Salti da un compito all'altro prima di finire il primo? Hai problemi di costanza? Cominci progetti, ma hai problemi a portarli a termine?
0 1 2

c. Ricordare oggetti, impegni
Tendenza a mettere nel posto sbagliato oggetti necessari per il lavoro o per la casa, a dimenticare i propri impegni (se questo problema è eliminato da un'attenta organizzazione, attribuire un punteggio pari a "0")

Sei sbadato? Ti dimentichi di richiamare qualcuno che ti ha chiamato o di rispettare gli appuntamenti? Smarrisci spesso oggetti come chiavi, borsa, portafoglio, o lasci le cose in giro per la casa o a lavoro?
0 1 2

d. Procrastinazione, evitamento
Fa scelte errate riguardo ciò che dovrebbe e ciò che non dovrebbe essere rimandato

Hai problemi legati al cominciare le attività, rimandarle, procrastinare? Lasci tutto per l'ultimo minuto? Fai fatica a rispettare le scadenze?
0 1 2

**7. Impulsività:** manifestazioni minori includono parlare prima di aver riflettuto, interrompere le conversazioni degli altri, l'impazienza (per es. alla guida), spese impulsive. Manifestazioni maggiori possono essere simili a quelle osservate nella mania o nel Disturbo Antisociale di Personalità e includono, in varia misura, una scarsa performance sul lavoro, l'iniziare o terminare bruscamente le relazioni (per es. multipli matrimoni, separazioni, divorzi), comportamenti antisociali come far le corse con la macchina, il taccheggio, l'eccessivo coinvolgimento in attività piacevoli senza riconoscere i rischi di conseguenze dolorose (per es. shopping compulsivo, folli investimenti economici, guida spericolata). Il soggetto prende le decisioni rapidamente e facilmente senza riflettere, spesso sulla base di informazioni insufficienti, a proprio svantaggio. Incapacità di rimandare l'azione senza sperimentare disagio.	**7. Impulsività**<br>**Punteggio riassuntivo 0-4:**<br>0 1 2 3 4
**a. Decisioni impulsive**	Hai problemi legati all'essere impulsivo? Ti butti spesso nelle cose senza pensarci? Prendi decisioni improvvise senza riflettere?<br>0 1 2
**b. Impulsivo verbalmente**<br>Spara risposte prima che le domande siano completate, è inopportuno	Sei impulsivo nel parlare? Interrompi gli altri? Finisci le frasi agli altri? Dici le cose senza pensare, le spari? Ti penti spesso delle cose che hai detto?<br>0 1 2
**c. Impulsivo col denaro**<br>Provoca problemi finanziari	Sei impulsivo con i soldi? Compri impulsivamente o hai difficoltà a gestire i soldi?<br>0 1 2
**d. Problemi con i dettagli/errori di distrazione**<br>La precisione è sacrificata per la velocità, gli è difficile controllare il lavoro	Sei frettoloso nelle attività o nel lavoro? Cerchi di fare le cose troppo rapidamente? Ignori spesso i dettagli? Fai errori di distrazione?<br>0 1 2
**e. Impazienza**<br>Fa fatica ad aspettare per il suo turno, non riesce ad attendere nelle code o nel traffico, si getta nelle cose di fretta saltando le istruzioni	Sei impaziente o incapace di aspettare? Gli altri ti vedono come impaziente (amici, familiari)?<br>0 1 2
